# Ordinary people, criminals, addicts and recreational users: Swedish court of law descriptions of persons sentenced for online drug purchases

**DOI:** 10.1177/14550725221079524

**Published:** 2022-03-29

**Authors:** Fredrik Tiberg, Johan Nordgren

**Affiliations:** 5264 Department of Social Work, Malmö University, Sweden; 5264 Department of Social Work, Malmö University, Sweden

**Keywords:** cryptomarkets, Darknet, digital capital, drug markets, drug offences

## Abstract

**Background:** The aim of this study was to analyze how Swedish courts describe persons sentenced for purchasing illicit drugs online. **Methods:** Qualitative analysis of naturally occurring data through 201 sentences that included 248 individuals sentenced for having purchased drugs online between January 1 2010 and January 1 2020. **Results:** The analysis resulted in the construction of four ideal types regarding the described characteristics of the sentenced persons; the ordinary person, the recreational user, the addict and the criminal. The courts operate with a notable dichotomy between traditional drug markets and online drug markets, that can be understood in relation to descriptions of Bourdieusian capital forms, specifically street capital and digital capital. **Conclusion:** Descriptions relating to street capital were of larger interest to the courts compared to digital capital, although there were examples of when the courts argued that uses of digital capital should be viewed as an aggravating circumstance. The courts largely held a dichotomous view of online and offline drug markets that focus on street-based criminality, which may have implications for how emerging digital drug markets are responded to by drug law enforcement and judicial systems.

The dynamics and nature of online drug markets is a recently established field of research interest in the Nordic countries (Bakken, 2021; Bakken & Demant, 2019; Bakken et al., 2018; Demant et al., 2020; Demant et al., 2019) as well as globally (Aldridge & Askew, 2017; Aldridge & Décary-Hétu, 2016; Koenraadt & van de Ven, 2018; Ladegaard, 2019; Moyle et al., 2019; Munksgaard, 2021; Scammell & Bo, 2016; Van Hout & Bingham, 2013). Online drug markets where illicit drugs and new psychoactive substances (NPS) are sold have been operating since the late 1990s and are a phenomenon that has grown in tandem with the increasing availability and use of the internet globally. Illicit drugs are sold in web shops on the surface web, via Darknet cryptomarkets (Orsolini et al., 2015) and through personal interactions on social media (Bakken, 2021; Moyle et al., 2019). Pharmaceuticals sold without prescription are most commonly traded on the surface web by sellers who describe themselves as “internet pharmacies” (Koenraadt & van de Ven, 2018; Scammell & Bo, 2016), while cryptomarkets are illicit drug marketplaces that use electronic currencies such as Bitcoin and cryptography to avoid law enforcement detection (Aldridge & Askew, 2017).

Online drug markets tend to be described by authorities as different to traditional drug markets in which buyers and sellers meet face to face to conduct transactions (Tollin et al., 2021). This dichotomy has been discussed through use of the theoretical concepts street capital and digital capital (Bakken & Demant, 2019; Sandberg & Pedersen, 2011), that are developments of Bourdieu's praxis theory (Bourdieu, 1980). While street capital is valuable in traditional street-level drug markets through skills needed to conduct drug transactions, digital capital is valuable in online drug markets where elements such as digital marketing, encryption and digital communication are needed to reach customers and avoid law enforcement (Bakken & Demant, 2019). Although research on online drug markets has recently been developed, we have found no previous study that has analysed how courts of law work with cases of online drug purchases. In this study we have conducted a qualitative analysis of sentences of persons who had bought illicit drugs online processed by Swedish courts between 2010 and 2020.

Although sentences for online drug sales are not a new phenomenon in Sweden, the development of Darknet markets, encryption and digital currencies such as Bitcoin imbue judicial and media discourses with the sense that online drug markets have become increasingly difficult to combat (The Swedish Police Authority, 2018). There is also a sense that online drug markets attract new populations of people who use drugs that previously have not been in contact with traditional drug markets. Some data suggest that Swedish Darknet cryptomarkets may attract customers with better socioeconomic circumstances than customers in traditional drug markets (Tollin et al., 2021). In the context of the Swedish drug policy approach this is viewed as increasing drug problems in society. Since Swedish drug policy has a strong emphasis on repression (Tham, 2005) it is of interest to investigate how online drug offences are handled in courts of law as well as whether persons sentenced for online drug offences are described as a new population of people who use drugs.

The aim of the study was to investigate how the Swedish courts describe persons sentenced for purchasing illicit drugs online. How courts write sentences and describe the people who are sentenced indicates how the judicial system handles emerging drug markets in relation to drug law enforcement, and may also shape public views on drug use and people who use drugs through their power to define deviance. The study was guided by the following research questions: (1) How are persons sentenced for purchasing illicit drugs online described by Swedish courts? and (2) In which ways do descriptions of the sentenced people relate to street and digital capital?

Analysing the characteristics of a large sample of persons who have been sentenced for buying drugs online adds to knowledge of the dynamics of online drug markets, as well as to the understanding of how these offences are handled within the judicial system. Considering the extent of drug law sentences annually processed by the Swedish court system, we have focused specifically on drug crimes facilitated online. We do not compare street-level drug crimes with online crimes, but we theorise about the often-made dichotomy between these market modalities. One important issue is thus to investigate how persons sentenced for online drug purchases are viewed within the judicial system and how we can understand how this system handles the emergence of new developing drug markets. Another issue concerns how the judicial system may contribute to shape discourses about people who use drugs and how its handling of criminality and notions of “addiction” can serve to further stigmatisation and discrimination (Spivakovsky & Seear, 2017). In the following section we describe the Swedish drug laws to contextualise the judicial system under study.

## Swedish drug laws

Sweden has two different legislations that regulate psychoactive substances. The Penal Law on Narcotics criminalises substances classified as narcotics and the Prohibition of Certain Goods Dangerous to Health Act criminalises substances not classified as narcotics but considered a danger to health and life (Träskman, 2011). The Penal Law on Narcotics contains four different classifications of crime: minor drug offence, normal drug offence, serious drug offence and exceptionally serious drug offence. The minimum sentences for drug offences in Sweden are unit fines, and the maximum is an 18-year prison sentence. Unit fines are reserved for minor drug offences. Punishment for violation of the Prohibition of Certain Goods Dangerous to Health Act ranges from unit fines as a minimum to a maximum one-year prison sentence (EMCDDA, 2019). Besides the legislation on drug offences, there is legislation regarding the smuggling of drugs, with the same classifications as the drug law (Träskman, 2011).

The courts’ choice of sanction is determined by the penal value of the crime and the personal circumstances of the sentenced individual. For drug offences, three main factors determine the penal value: the amount of the drug, the type of drug and other circumstances; for example, whether the sentenced person intended to resell the drug (Träskman, 2011). Drug offences are regarded as “character crimes” by the Swedish court system, which means there is a strong presumption of imprisonment. Therefore, a prison sentence is the primary sanction for drug offences that are any more serious than a minor offence. Sanctions without deprivation of liberty must be motivated by referring to mitigating personal circumstances of the sentenced person (Jareborg & Zila, 2017). Personal circumstances can be both mitigating and aggravating factors in the court's choice of sanction. Drug addiction and mental health problems, as well as the absence of a previous criminal record, are seen as mitigating circumstances, while recent convictions for similar crimes are considered aggravating circumstances (Jareborg & Zila, 2017).

Swedish law has nine different sanctions and in total 17 different combinations of sanctions. The sanctions are unit fines, conditional sentences, community service, probation, youth care, youth service, institutional care of young persons, prison and forensic psychiatric care. Unit fines are the mildest sanctions, and prison sentences the most serious. Other sanctions are not ordered in a hierarchy but are equivalent to a prison sentence. The prosecutor can also decide to waive prosecution, which is a sentence without a sanction (Jareborg & Zila, 2017).

## Previous research

Previous research on sales of illicit substances on the internet has generally used three types of methods: online surveys, web monitoring and interviews (Orsolini et al., 2015). Research into cryptomarkets on the Darknet is a research field that has grown since the emergence of the Silk Road cryptomarket in 2011 (Ladegaard, 2019; Orsolini et al., 2015). Demographically, the customers of cryptomarkets are described as men in their 20s, the majority of them being employed or students (Barratt et al., 2016; Van Hout & Bingham, 2013) and they are generally described as recreational drug users (Orsolini et al., 2015).

Cryptomarkets operate differently to traditional drug markets, since trust is not mainly built upon personal relationships. The anonymity of cryptomarkets enables vendors to advertise their products, and buyers to provide public feedback (Bakken et al., 2018). In cryptomarkets, the operators also act as third parties to resolve disputes; for example, regarding customer complaints about orders not delivered (Tzanetakis et al., 2016). The structure of the cryptomarkets reduces the problems of security and visibility that exist in traditional drugs markets, which might lead to new populations of vendors and purchasers of drugs. Because of this, cryptomarkets could increase access to psychoactive substances (Bakken et al., 2018). Customers perceive cryptomarkets as safer than traditional open drug markets, with less perceived risk of violence and detection (Barratt et al., 2016) and as offering better customer service and drugs of better quality compared to traditional drug markets (Ormsby, 2016).

It is common for customers of cryptomarkets also to purchase drugs from friends, dealers and, to a lesser degree, open drug markets (Barratt et al., 2016). Aldridge and Décary-Hétu (2016) studied the revenue from substances sold in larger quantities on Silk Road, concluding that 25% of the revenue on Silk Road was generated by purchases for resale. Cryptomarkets supply not only individual consumers of illicit drugs, but also local traditional drug markets, and they thereby supply illicit drugs to a wider group than their first-hand customers (Aldridge & Décary-Hétu, 2016).

The substances most commonly sold in cryptomarkets reflect those sold in traditional drug markets to some extent. MDMA and stimulant-type substances such as synthetic cathinones are more common in cryptomarkets, and opioids are more common in traditional drug markets (Kruithof et al., 2016), although fentanyl analogues continue to be sold in cryptomarkets (Lokala et al., 2019). There is also evidence that cryptomarkets react to law enforcement crackdowns similarly to traditional drug markets, in that vendors relocate to other Darknet marketplaces (Ladegaard, 2019).

Research into the sales of medicines without prescription via web shops on the surface web is limited and focuses mostly on the characteristics of the consumers and sellers of such pharmaceuticals. The purchasers are generally described as a more heterogeneous group than the purchasers who use cryptomarkets, but as predominantly male between 20 and 40 years old (Orsolini et al., 2015). Koenraadt and van de Ven (2018) have studied the demographic characteristics and motives of purchasers of “lifestyle drugs”, taken to improve lifestyle, health or beauty. The study estimates that 1.6% of the Dutch population had at least once purchased medicines such as Viagra or Ritalin online. As with cryptomarkets, some of the pharmaceuticals bought on the surface web are bought in larger quantities and resold in local drug markets (Scammell & Bo, 2016).

The sale of illicit substances on social media has recently become a focus of increased research, which mainly analyses the interaction between sellers and customers (Demant et al., 2020; Moyle et al., 2019). Social media apps are often used for initial contact, and once the contact is established, the communication is switched over to encrypted messaging apps such as Wickr. The exchange of drugs for money is generally made in person and to a lesser degree by mail, and it is common for social media drug dealers to purchase their drugs from cryptomarkets (Demant et al., 2019).

## Methods

To study how the Swedish justice system describes people sentenced for purchases of illicit drugs online, we employed a qualitative approach in our analysis of sentences. The empirical material consists of sentences given by Swedish district courts and courts of appeal. Court documents such as sentences are public records in Sweden and available to all citizens through the Public Access to Information and Secrecy Act. As such, the material consists of naturally occurring data that can be used in order to gain new insights into drug use in society (Enghoff & Aldridge, 2019). Research that uses documents as source materials needs to take the specific nature of these documents into consideration. John Scott (1990) argues that four principles are central to document analysis: authenticity, credibility, representation and meaningfulness. When interpreting documents, it is important to note that they do not just describe events, organisations and individuals. Documents have a specific function within the organisation where they are created, with specific terms and language. Since documents have specific functions, they also have the rhetorical function of creating accounts that construct versions of reality. This means that documents cannot be treated as firm evidence of what they report, regardless of their official status. In the analysis of documents, reflexivity is required of the researcher in understanding the limitations and possibilities of the documents. According to Coffey (2014), it is more fruitful to ask questions about the form and function of a document rather than about its truthfulness. Sentences are documents that have the rhetorical function of arguing for the sentencing of the accused as guilty or not guilty, and for the justification of a specific sanction.

The district courts are the courts of first instance for the proceedings of criminal cases in Sweden. If the case is appealed, the second instance for criminal cases is the Court of Appeal, with the Supreme Court being the last instance, which is generally reserved for cases of presidential value (Jareborg & Zila, 2017). The sentences are a documentation of the process of the trial and are written by the judge who also is the chairman of the trial. The first part of the sentences consists of a summon of the verdict, the name of the accused, the classification of crime and the sanction. The second part of the sentences is a summary of the main hearing of the trial in which the prosecutors defend the prosecution of the accused person by presenting evidence from the criminal investigation and testimonies from witnesses which are responded to by the accused persons and the defence attorney. The personal circumstances of the accused person are also investigated through hearing the accused but also by the personal investigation of the Probation Service. The following part of the sentence consists of an argument by the prosecutor for the guilt of the accused and a proposal of sanction which is answered by the defence attorney. The verdict is the last part of the sentence in which the court presents why they find the accused guilty or not based on the evidence presented during the trial. Since our sample only includes convicting sentences, the sentences also include the courts’ argument on the penal value of the offence, which in drug cases mainly is determined by the type and amount of drug seizure. However, aggravating or mitigating circumstances regarding the offence may also affect the penal value. The courts also argue for a sanction based on the penal value of the offence and the personal circumstances of the sentenced person.

The sentences were acquired through the juridical database Juno, owned by the Karnov Group. The keywords used for searching the database were “narcotics” and “internet” (in Swedish *narkotika* and *internet*). In total, 6,089 hits were found in the database from 1 January 2010 to 1 January 2020. The summary and crime classifications of all the hits were investigated for relevance to the study. The sample criteria used were that the sentenced person had bought illicit drugs on the internet, according to the statement of the criminal act given in the sentence, or that the accused had confessed to buying the substances on the internet and that a parcel had been confiscated. After careful reading, 201 sentences that met the sampling criteria remained, including in total 248 sentenced individuals. The sentences vary in length from six pages up to 120 pages. Of the 201 sentences, 140 were from district courts and 61 were from courts of appeal. None of the sentences from the Supreme Court met the sampling criteria. Since courts of appeal are higher-tier courts than the district courts, the sentences from the courts of appeal had precedence when duplicates were found. According to Karnov, the database includes all sentences from the Supreme Court and Court of Appeal but not all sentences from the district courts (Karnov Group, personal communication, 2019). Minor offences are likely underrepresented in the sample, while serious drug offences are likely overrepresented since they are more likely to be appealed.

Our qualitative analysis uses ideal types as a theoretical tool to investigate how the sentenced persons and their motives are described in the sentences. The purpose of ideal types is to create models of groups or processes to better understand certain phenomena in society. The researcher concentrates on certain elements of the studied phenomena, which means that the ideal type is not a representation of reality or real persons (Rosenberg, 2016). According to Swedberg (2018), ideal types can be used for different purposes: either to create new terminologies or classifications or to use heuristics to develop and explore new ideas about social reality. In this study we use ideal types heuristically in our analysis of the empirical material.

The first step in the coding of the material was to carefully read the sentences to obtain an overview. It became apparent that descriptions of character traits and social circumstances of the sentenced persons were diverse. The differences in these descriptions were primarily in how the sentences described the social circumstances and motives of those sentenced. The courts use descriptions of the social circumstances of the sentenced persons to explain the cause of the offence and to argue for the choice of a sanction. The coding of social circumstances was therefore used to obtain information on how the cause of the offence was described. Social circumstances were primarily defined in relation to employment, housing and personal relationships. The social circumstances of the sentenced persons could be separated into two groups, in which the sentenced person was described as ordered or disordered.

In the sentences, the described motive of the sentenced person was also used by the courts to explain his or her actions and to argue for a sanction. The motives could be separated into four differing categories: mitigation of personal problems, addiction, profit and personal experimentation. Based on the patterns of description of motive and social circumstances, four ideal types were created. The ideal types are based on descriptions of motive, social circumstances and the courts’ choice of sanction and motivation for ordering it. The empirical material was coded based on the ideal types using NVivo Version 12. Excerpts from the sentences that exemplify the ideal types were selected and translated from Swedish into English by the authors.

The study has been approved by the Swedish Ethical Review Authority (Dnr. 2020-03320). To avoid identification of individuals we have given them pseudonyms, and we have altered or removed places of residence, dates and changed specific types of drugs to the general group of substance or to another one in the same group.

## Results

The sentenced persons are a diverse group in relation to age, geography, the substance they bought and their previous criminal records. They are represented in all types of cities in Sweden in terms of size. Although they constitute a diverse group, the typical sentenced person is a male in his early 30s sentenced for drug smuggling to a sanction without deprivation of liberty. Among the sentenced individuals, the purchase of pharmaceuticals and illicit drugs was more common than the purchase of NPS. The five most common substances purchased were benzodiazepines, tramadol, amphetamine, cannabis and synthetic cathinones.

In the justice system, a narrative is created regarding the accused and sentenced person. In the creation of the narrative of the crime, the sentenced person, the Probation Service, the police and the court are contributors. Descriptions of the social circumstances and motive of the sentenced person creates a story of why he or she committed the offence. The social circumstances of the sentenced persons could be separated into two groups, in which the sentenced person was described as ordered or disordered in relation to housing, employment, social network and alcohol and drug use. The courts describe unemployment, homelessness, a social network with criminal values and drug addiction as disordered social circumstances. Our analysis of the material resulted in the creation of four different ideal types: the ordinary person, the addict, the criminal and the recreational user, which we present in the following sections.

### The ordinary person

The ordinary person ideal type as described by the courts is a person living under ordered circumstances, but who encounters problems in life such as a workplace accident or family problems, and has purchased drugs or pharmaceuticals as a reaction. He or she is generally cooperative toward the justice system. The following excerpt from a Probation Service pre-sentencing report describes a man sentenced to probation for a normal drug offence for over 40 purchases of 20 pain medication pills on the surface web:

From the information Hans Nilsson provided and /…/ the investigation material, he seems to live in orderly and good circumstances. Nilsson admits the facts [of the crime] and it is not possible to identify criminal values ​​or attitudes.

The Probation Service statement that Hans lives under “good circumstances” is worth noting since it is a positive description, and it denotes an ascription of some degree of economic and social capital. The lack of criminal values or attitudes is another characteristic of the ordinary person. In general, the ordinary person has no previous criminal record. In descriptions of an ordinary person's description of him- or herself there is no identification as a criminal, as exemplified in the excerpt below:

Daniel has ordered the substances anonymously on the internet and paid with bitcoins. /…/ Daniel has no connections in the underworld and thinks it's easier to order online.

Daniel was sentenced to probation for drug smuggling when he bought 100 grams of central stimulants through a Darknet cryptomarket and is described as having no connections to the “underworld”. He argued that he was part of mainstream society and not of a criminal subculture; however, Daniel's statement regarding a lack of connections to “underworld” criminality was not mentioned in the verdict. The ordinary person is, in general, not described as possessing digital capital and it is more common for him or her to have purchased pharmaceuticals from web shops on the surface web, which requires less technical knowledge compared to Darknet markets. The ordinary person's lack of collective identity in relation to their use of illicit drugs is also characteristic of descriptions of this type. For those who have purchased pharmaceuticals, the motivation for the purchase is explained by their relationship with physicians and health services and not with drug markets (cf. Koenraadt and van de Ven, 2018), as exemplified in the quotation below, which describes a man sentenced to probation for drug smuggling for having purchased 400 sleeping pills on the surface web:

Claes has previously had “Theralen” and “Zolpidem” [an antihistamine and a benzodiazepine analogue] prescribed by a Swedish doctor. Since those tablets did not suit him, he wanted to try something different. Swedish doctors do not prescribe other medicines. To avoid going to the Swedish doctor again he ordered sleeping pills on the internet.

The ordinary person's use of illicit drugs is described as a form of self-medication to handle everyday life and to mitigate problems, not to experience euphoria or mind alteration. In the excerpt below follows a description of a man who had bought 2000 pills of tranquilizers and was given a conditional sentence with community service for a minor drug offence:

Mats said that he had Xanax on prescription and then noticed that using the pills made it easier to cope with everyday life. He had a difficult time with problems in his relationship and deaths in the family. He started ordering pills on a website. His addiction escalated, and he bought more and more.

The ordinary person's desire to handle various problems in life is described by the courts as a mitigating circumstance, as are the lack of previous criminal records and the ordered social circumstances. However, these mitigating circumstances have a limited effect on the penal value of the offence if it is a minor drug offence (Träskman, 2011), although the courts take them into account when choosing a sanction for the offence. The courts’ sentences for the ordinary person, therefore, are primarily sanctions without deprivation of liberty, such as probation.

### The addict

The addict ideal type is described as having disorderly social circumstances in relation to several areas of life, such as mental health, crime, employment and social relations. In terms of capital forms, the addict is ascribed low levels of economic and social capital. Although the term “addict” is a stigmatising ascription of identity (Singer & Page, 2014), we use it here as an ideal type based on the descriptions found in the empirical material. The Swedish term most used in the material is “missbrukare”; a term that is often translated into English as “addict” or “drug abuser”.

The addict is cooperative in relation to the justice system and often confesses to the crime. His or her use of illicit drugs is described as emerging when he or she was a juvenile or in early adulthood. The addict has often previously been sentenced, particularly for drug offences, and has served a prison sentence. Even though the addict is described as a person with problems regarding criminality, this criminality is primarily viewed as a consequence of the drug use. The following sentence describes the case of a man who was sentenced for drug smuggling to a prison sentence, for purchasing 100 grams of synthetic cathinones on the surface web. Mikael framed his motivation for using online drug markets as resulting from his dissatisfaction with other drug markets:

Mikael is a drug addict, but at the same time he wants to keep a low profile. /…/ For him it was all set and done, very simple, and he saw just how everything was going to be solved for him, not having to interfere with drug addicts and problems in society; he saw it as an opening to a simpler life for him.

Mikael used online drug markets because he did not want to get involved with other “drug addicts” to purchase illicit drugs. In the excerpt it is also clear that Mikael is described as having a subcultural collective identity as a drug addict, which is also characteristic of this ideal type. An economic motive is a common motive for purchasing drugs on the internet for the addict ideal type. Below is an excerpt from the sentence of a man sentenced to probation for drug smuggling after having bought 100 grams of amphetamine from the Netherlands:

Nicklas was feeling very bad in 2015 and started using more and more drugs. Initially, he bought the drugs on the street but it became too expensive, so he started ordering from the internet instead. /…/ He ordered 100 grams of amphetamine at a ridiculously cheap price. He paid |SEK 2,500 for the amphetamine, which at home on the street would have cost between SEK 10,000–12,000.

The purchase of drugs from online drug markets became a solution for Nicklas's escalating and expensive drug use. To purchase drugs on the internet was not just cheaper, it was “ridiculously cheap” compared to purchasing drugs on the street in Sweden. To be able to purchase drugs so cheaply otherwise, Nicklas would have to purchase them directly from drug markets abroad.

Besides the penal value, two aspects are central when the courts choose sanctions for the offences of the addict. Is the addict motivated to change and does he or she need any kind of treatment for drug abuse or mental health problems? The criminality of the addict is considered to be primarily caused by the addiction. Since the addict is seen as lacking control over his or her behaviour, the authorities need to supervise and control the addict, preferably through treatment. The addict's personal circumstances and conduct allow the court to justify treatment instead of prison if the offence is not too severe in relation to the treatment required.

### The criminal

The criminal ideal type is characterised by being described as a criminal primarily, even though they may have problems with drug addiction or mental health problems. The criminal is generally not cooperative with law enforcement and the justice system. In sentences concerning the criminal, it is common that several people have been involved in the crime and that the purchase of illicit drugs was with intent to resell. In the excerpt below, the court describes the profit motive of a man sentenced for a serious drug offence to a prison sentence for purchasing 200 grams of central stimulants through a Darknet market in order to resell them on social media platforms:

Finally, while Markus Karlsson's crime has to some extent been associated with his own abuse, it seems that the decisive purpose of the sale has been to make money. /…/ He has thus spoken of how he saw the sale of drugs as a “livelihood”.

The courts view the motive of profit-making and the organisation behind the offence as aggravating circumstances. The criminal has been sentenced to a stay in prison before, and many have an extensive criminal record. The criminal's motive for purchasing drugs online is primarily profit, by purchasing substances more cheaply abroad and reselling them in local drug markets, indicating a high degree of street capital. Online drug markets offer the opportunity of drug smuggling to people who cannot purchase larger quantities of drugs in drug markets abroad. Both Darknet cryptomarkets as well as shops on the surface web are commonly used by the criminal. The drugs are either resold in traditional drug markets or in social media and domestic cryptomarkets, which means that the digital capital among the criminal ideal type could be described as varying from rudimentary to high level. The use of the internet to sell drugs is considered an aggravating circumstance by the Swedish courts. One of the sentences regarding a person who has given a prison sentence for reselling one kilo of central stimulants in a domestic cryptomarket argues that this was a particularly dangerous offence:

It is also a question of activities that are difficult to detect, and which allow the distribution of drugs to a large number of people in a relatively simple way. Thus, the operations have been of a particularly dangerous nature. /…/ The district court argues that it is reasonable to assume that the buyers were young people who have a good knowledge of computers, software and the internet and who have not yet developed a heavy drug addiction.

When the courts sentence the criminal ideal type, the only sanction available is a prison sentence, primarily because of the penal value of the offence, which often involves larger quantities of illicit drugs. The criminal's motive of profit through the resale of the drugs purchased online is an aggravating circumstance, as well as the criminal's cooperation with other criminals. The intent of the criminal to make profit makes the mitigating circumstances of addiction and mental health problems irrelevant since they are not considered as influencing the intention of the crime.

### The recreational user

Descriptions of the recreational user ideal type are characterised by the individual's search for euphoric experiences and self-exploration, in a way that is found in psychonaut subculture (Söderberg, 2016). The motive of the recreational user differs from that of the other ideal types by the desire for positive experiences rather than the need to mitigate problems or obtain economic profit. The following excerpt concerns a younger man sentenced to a conditional sentence for the purchase of 30 grams of hallucinogenic mushrooms on the surface web:

Gabriel Johansson has stated the following: he had an idea about trying [psilocybin] mushrooms because he heard that they could give a new perspective on the world and be an interesting experience. He ordered the mushrooms from an internet site.

The recreational user's drug use is described as sporadic rather than a regular habit, and he or she does not view the drug use as criminal or problematic. The Probation Service describes the drug use of one recreational user in the excerpt below:

Martin Olsson has used hashish at least once a month for the past six months and justifies his use by saying that this is something everyone does who has been at a party. The Probation Service considers that there is a recidivism risk and a need for supervision, but states that Martin Olsson does not consider himself in need of any support and assistance from the Probation Service.

Martin's view that drug use could be unproblematic conflicts with the Probation Service discourse on drugs. The Probation Service represents the restrictive Swedish drug policy in which all use of illicit drugs is considered a form of “drug abuse” (Edman & Olsson, 2014). What also characterises the recreational drug user is a collective identity as a user of drugs as opposed to someone who abuses drugs. The recreational user lives under ordered social circumstances, and neither mental health problems nor criminality apart from the drug use are mentioned in the sentences. He or she rarely has a criminal record, and if there is such a record, it is typically for a minor drug offence. However, the pursuit of euphoric and new experiences does not constitute an acceptable justification or excuse for the courts. The recreational user does not express criminal values besides the justification of drug use, and his or her ordered social conditions and lack of a criminal record speaks to the user's advantage.

## Discussion

In this study we have analysed how Swedish courts describe persons sentenced for purchasing drugs online. Our qualitative analysis of sentences allowed us to identify the four different ideal types that we constructed on the basis of how the sentenced persons were described in terms of social circumstances and motives. Sentences are documents that have a rhetoric function (Coffey, 2014), which means that the descriptions of the sentenced persons also have the rhetoric function of arguing for a sanction. It is therefore likely that the sanction also affects how the sentenced person is described. The analysed sentences contain language use that may promote stigmatisation and discrimination of people who use drugs, such as in the addict ideal type. As argued by Spivakovsky and Seear (2017) in their study of drug courts, sweeping generalisations about people described as “addicts” may result in problematic depictions regarding abnormality, dangerousness and propensity for criminal behaviour.

We find that Swedish courts mostly operate with a dichotomous comparison between online and offline trading when handling cases of online drug purchases. This dichotomy has been noted by Barratt in research on cryptomarkets, and is notable also in Swedish authorities’ approaches to drug markets (Barratt et al., 2016; Tollin et al., 2021). Pierre Bourdieu's praxis theory has been employed in a wide range of research fields to explain the various ways in which people act in relation to social structures, notably regarding people who may use or sell drugs in specific subcultures (Bakken & Demant, 2019; Bourdieu, 1980; Sandberg, 2008; Sandberg & Pedersen, 2011). The basis of this theory is that society is constituted by different social fields with specific hierarchies and norms. Individuals acting within fields can employ different forms of capital, such as economic, social, cultural, and symbolic. These forms of capital can be used by individuals to navigate in the field in relation to other actors. A central point is that capital types can be exchanged into other types based on which capital is valued in the specific field. For example, economic capital can be transferred to social, cultural or symbolic capital, but this process is costly in terms of time, money and effort (Bourdieu, 1980).

Sandberg and Pedersen (2011) have developed the Bourdieusian analysis by introducing the notion of street capital, which is a form of cultural capital employed in open drug markets. Street capital is based on a culture outside of mainstream society, with its own rules, norms and hierarchies. When acting in open drug markets the individual's street capital accumulates over time. Street capital is useful for marginalised people with limited social, cultural and economic capital in mainstream society, for whom street capital offers opportunities of recognition and income. With the development of online drug markets, researchers have turned their attention to which kinds of capital are valued in such markets. One issue concerns access to online drug markets. Drug markets in general may be characterised as open or closed to the general public (May & Hough, 2004). Online pharmacies on the surface web are open to everyone and do not necessitate personal relationships between vendor and purchaser. However, Darknet markets are closed in the sense that digital know-how is needed to access them, and sometimes also relationships to other users in order to find specific marketplaces. Access to and engagement with Darknet markets thus require a high degree of digital capital, as suggested by Bakken and Demant (2019). In this case, digital capital is the technical knowledge of encryption and the ability to act according to the scripts of the digital drug trade. Although digital capital is specific to the digital drug markets, it has more similarities to what is valued in mainstream society since it puts an emphasis on well-written communication, marketing and technical knowledge (Bakken & Demant, 2019). Possession of digital capital may also be combined with street capital. Sales of illicit drugs on social media often require a combination of the two, with digital capital required to market the drugs on social media platforms and street capital required in the transaction, since the drugs are exchanged through personal meetings (Bakken & Demant, 2019). For example, the criminal ideal type may lack access to drug markets abroad and he or she can employ street capital to sell drugs in local drug markets, or digital capital to sell drugs in domestic online drug markets.

The ways the sentenced persons are described by the courts show some patterns that relate to the identified ideal types. The criminal has the highest degree of ascribed digital capital and street capital among the ideal types, while the ordinary person has the least degrees of these two types. Perhaps this is explained by the social circumstances and the motives of the ideal types – since the ordinary person possesses economic and social capital, they may be less motivated by the benefits of accumulating street and digital capital since their main motive is to cope with personal difficulties in life. The criminal, however, is motivated by the benefits of accumulating both street capital and digital capital to be able to sell drugs and gain profit. Since the exchange of capital forms is costly in terms of time, money and effort (Bourdieu, 1980) the individual needs to be motivated to accumulate symbolic capital in relation to a new field such as Darknet cryptomarkets. The criminal may also increase his or her street capital by purchasing drugs in online drug markets and reselling them in traditional domestic drug markets. The recreational user employs both Darknet cryptomarkets and shops on the surface web and is described as having a low degree of street capital and having an ordered social situation with social and economic resources. The main motive of the recreational user is the search for new experiences, which motivates him or her to accumulate digital capital to access the wider variety of substances available through online drug markets (Ormsby, 2016). The drug addict lacks social and economic capital but also to some degree street capital to access satisfying drug markets. The addict also has an extensive and costly drug use which motivates him or her to accumulate digital capital to access drugs at lower prices from online drug markets.

Aspects relating to street capital seem to be of greater concern to the courts compared to digital capital, although we also found some consideration of digital capital in terms of reselling drugs procured online to offline markets. Digital capital, however, has not been of primary interest to the Swedish courts. It is, rather, the sentenced persons’ relation to street capital that has been of primary interest in the documents. The possession of street capital is viewed as an aggravating circumstance by the courts, while the lack of street capital is viewed as a mitigating circumstance. The street capital of the sentenced person is particularly of interest to the courts when the sentenced person has purchased drugs online to resell via local drug markets. The digital capital of the sentenced person is described as a means to increase street capital in those sentences where drugs were purchased in larger quantities from abroad and resold for profit.

The only sentences where digital capital is of primary interest are those where the sentenced person resold the purchased drugs through domestic online drug markets. The courts regard digital capital among sellers of drugs as an aggravating circumstance, since digital capital enables the purchase of drugs by persons who lack the criminal connections and street capital to purchase drugs, while digital capital among sentenced persons who only purchase drugs on the internet is considered neither a mitigating nor an aggravating circumstance by the courts. Street capital is possibly of greater concern in the court system, since it is considered an expression of criminal values and a cause of criminal actions.

The descriptions of the motives for purchasing drugs online in the sentences can be divided into two types: the inability to access traditional drug markets or a dissatisfaction with the drug markets available to the individual. Online drug markets are described as offering different customer relationships, a better variety and quality of drugs and lower prices (Barratt et al., 2016). The motive relating to different customer relationships is primarily based on a notion that online drug markets offer the possibility of a more professional relationship between purchasers and sellers of drugs compared to traditional drug markets. The motives of purchasers of pharmaceuticals without prescription were often linked to the user's inability to obtain a desired or needed prescription from doctors or the healthcare system (cf. Koenraadt and van de Ven, 2018). This group may view themselves as users of medications and not as users of illicit drugs and therefore prefer using web shops that describe themselves as online pharmacies, rather than using traditional drug markets, considering their relative lack of ascribed digital capital.

### Strengths and limitations

Our study has provided analysis of a large set of naturally occurring data spanning ten years, which provide new knowledge of how a national court system handles crime relating to an emerging drug market structure. The nature of the data has a limitation in that the sentences describe the aftermath of the judicial process and do not capture the interactional processes occurring in court (cf. Elsrud et al., 2017). The identified ideal types are used heuristically in our analysis and our study cannot answer to what extent or in what ways these are put to active use in the judicial processes about online drug purchases.

## Conclusions

Our study allowed us to identify four different ideal types of persons sentenced for having purchased drugs through both open and closed online markets. The ideal types seem to reflect the Swedish drug policy approach that defines all non-prescribed drug use as illicit “drug abuse”, and language used in the sentences may further stigma toward people who use drugs. We found descriptions relating to street capital to be of greater interest to the courts compared to digital capital. However, we also found examples of when the courts argued that uses of digital capital should be viewed as an aggravating circumstance. The courts largely operate with a dichotomous view of online and offline drug markets that focuses on street-based criminality, which may have implications for how emerging digital drug markets are responded to by drug law enforcement. Our study suggests that the online/offline dichotomy is of limited value in general, but that a consideration of both street and digital capital among drug law offenders may be beneficial in understanding current drug market dynamics. The present study concerns people who have been sentenced for purchasing drugs from online markets, while further research might focus on how the courts handle cases of vendors who use online drug markets.

## References

[bibr1-14550725221079524] Aldridge J. Askew R. (2017). Delivery dilemmas: How drug cryptomarket users identify and seek to reduce their risk of detection by law enforcement. International Journal of Drug Policy, 41, 101–109. 10.1016/j.drugpo.2016.10.010 28089207

[bibr2-14550725221079524] Aldridge J. Décary-Hétu D. (2016). Hidden wholesale: The drug diffusing capacity of online drug cryptomarkets. International Journal of Drug Policy, 35, 7–15. 10.1016/j.drugpo.2016.04.020 27260863

[bibr3-14550725221079524] Bakken S. A. (2021). Drug dealers gone digital: Using signalling theory to analyse criminal online personas and trust. Global Crime, 22(1), 51–73. 10.1080/17440572.2020.1806826

[bibr4-14550725221079524] Bakken S. A. Demant J. (2019). Narkotikamarkeder på nett: En begrepsutvikling av digitalkapital [Drug markets online: A conceptual development of digital capital]. Norsk sosiologisk tidsskrift, 3(3), 209–227. 10.18261/issn.2535-2512-2019-03-04

[bibr5-14550725221079524] Bakken S. A. Moeller K. Sandberg S. (2018). Coordination problems in cryptomarkets: Changes in cooperation, competition and valuation. European Journal of Criminology, 15(4), 442–460. https://doi.org/10.1177%2F1477370817749177

[bibr6-14550725221079524] Barratt M. J. Ferris J. A. Winstock A. R. (2016). Safer scoring? Cryptomarkets, social supply and drug market violence. International Journal of Drug Policy, 35, 24–31. 10.1016/j.drugpo.2016.04.019 27241015

[bibr7-14550725221079524] Bourdieu P. (1980). The logic of practice. Stanford University Press.

[bibr9-14550725221079524] Coffey A. (2014). Analysing documents. In Flick U. (Ed.), The SAGE handbook of qualitative data analysis (pp. 367–379). Sage Publications.

[bibr10-14550725221079524] Demant J. Bakken S. A. Hall A. (2020). Social media markets for prescription drugs: Platforms as virtual mortars for drug types and dealers. Drugs and Alcohol Today, 20(1), 36–49. 10.1108/DAT-06-2019-0026

[bibr11-14550725221079524] Demant J. Bakken S. A. Oksanen A. Gunnlaugsson H. (2019). Drug dealing on Facebook, Snapchat, and Instagram: A qualitative analysis of novel drug markets in the Nordic countries. Drug and Alcohol Review, 38(4), 377–385. 10.1111/dar.12932 31050051

[bibr12-14550725221079524] Edman J. Olsson B. (2014). The Swedish drug problem: Conceptual understanding and problem handling, 1839–2011. Nordic Studies on Alcohol and Drugs, 31(5–6), 503–526. https://doi.org/10.2478%2Fnsad-2014-0044

[bibr13-14550725221079524] Elsrud T. Lalander P. Staaf A. (2017). Noise, voice and silencing during immigrant court-case performances in Swedish district courts. Ethnicities, 17(5), 667–687. 10.1177/1468796815588620

[bibr14-14550725221079524] EMCDDA. (2019). Sweden country drug report 2019. Publications Office of the European Union.

[bibr15-14550725221079524] Enghoff O. Aldridge J. (2019). The value of unsolicited online data in drug policy research. International Journal of Drug Policy, 73, 210–218. 10.1016/j.drugpo.2019.01.023 30711411

[bibr17-14550725221079524] Jareborg N. Zila J. (2017). Straffrättens påföljdslära [Sanctions in criminal law]. Wolters Kluwer.

[bibr18-14550725221079524] Karnov Group. (2019). *Om domarna* [About the sentences]. Personal communication.

[bibr19-14550725221079524] Koenraadt R. van de Ven K. (2018). The internet and lifestyle drugs: An analysis of demographic characteristics, methods, and motives of online purchasers of illicit lifestyle drugs in the Netherlands. Drugs: Education, Prevention and Policy, 25(4), 345–355. 10.1080/09687637.2017.1369936

[bibr20-14550725221079524] Kruithof K. Aldridge J. Décary-Hétu D. Sim M. Dujso E. Hoorens S. (2016). Internet-facilitated drugs trade: An analysis of the size, scope and the role of the Netherlands. RAND Corporation.

[bibr21-14550725221079524] Ladegaard I. (2019). Crime displacement in digital drug markets. International Journal of Drug Policy, 63, 113–121. 10.1016/j.drugpo.2018.09.013 30572247

[bibr22-14550725221079524] Lokala U. Lamy F. R. Daniulaityte R. Sheth A. Nahhas R. W. Roden J. I. Yadav S. Carlson R. G. (2019). Global trends, local harms: Availability of fentanyl-type drugs on the dark web and accidental overdoses in Ohio. Computational and Mathematical Organization Theory, 25, 48–59. 10.1007/s10588-018-09283-0 32577089 PMC7311101

[bibr23-14550725221079524] May T. Hough M. (2004). Drug markets and distribution systems. Addiction Research & Theory, 12(6), 549–563. 10.1080/16066350412331323119

[bibr24-14550725221079524] Moyle L. Childs A. Coomber R. Barratt M. J. (2019). #Drugsforsale: An exploration of the use of social media and encrypted messaging apps to supply and access drugs. International Journal of Drug Policy, 63, 101–110. 10.1016/j.drugpo.2018.08.005 30530252

[bibr25-14550725221079524] Munksgaard R . (2021). Trust and exchange: The production of trust in illicit online drug markets [PhD thesis, The University of Montreal]. https://papyrus.bib.umontreal.ca/xmlui/handle/1866/25234

[bibr26-14550725221079524] Ormsby E. (2016). Silk Road: Insights from interviews with users and vendors. The internet and drug markets. EMCDDA.

[bibr27-14550725221079524] Orsolini L. Francesconi G. Papanti D. Giorgetti A. Schifano F. (2015). Profiling online recreational/prescription drugs’ customers and overview of drug vending virtual marketplaces. Human Psychopharmacology: Clinical and Experimental, 30(4), 302–318. 10.1002/hup.2466 26216567

[bibr28-14550725221079524] Rosenberg M. M. (2016). The conceptual articulation of the reality of life: Max Weber’s theoretical constitution of sociological ideal types. Journal of Classical Sociology, 16(1), 84–101. https://doi.org/10.1177%2F1468795X15574414

[bibr29-14550725221079524] Sandberg S. (2008). Street capital: Ethnicity and violence on the streets of Oslo. Theoretical Criminology, 12(2), 153–171. 10.1177/1362480608089238

[bibr30-14550725221079524] Sandberg S. Pedersen W. (2011). Street capital: Black cannabis dealers in a White welfare state. Policy Press.

[bibr31-14550725221079524] Scammell L. Bo A. (2016). Online supply of medicines to illicit drug markets: Situation and responses. The internet and drug markets. EMCDDA.

[bibr32-14550725221079524] Scott J. (1990). A matter of record. Documentary sources in social research. Polity Press.

[bibr44-14550725221079524] Singer M. Page J.B. (2014). The social value of drug addicts: Uses of the Useless. Routledge.

[bibr33-14550725221079524] Söderberg J. (2016). DIY research in the psychonaut subculture: A case of unwanted user innovation. In Hyysalo S. Jensen T. E. Oudshoorn N. (Eds.), The new production of users: Changing innovation collectives and involvement strategies (pp. 297–323). Routledge.

[bibr34-14550725221079524] Spivakovsky C. Seear K. (2017). Making the abject: Problem-solving courts, addiction, mental illness and impairment. Continuum, 31(3), 458–469. 10.1080/10304312.2016.1275152

[bibr36-14550725221079524] Swedberg R. (2018). How to use Max Weber’s ideal type in sociological analysis. Journal of Classical Sociology, 18(3), 181–196. https://doi.org/10.1177%2F1468795X17743643

[bibr37-14550725221079524] Tham H. (2005). Swedish Drug policy and the vision of the good society. Journal of Scandinavian Studies in Criminology and Crime Prevention, 6(1), 57–73. 10.1080/14043850510035128

[bibr38-14550725221079524] The Swedish Police Authority. (2018). Swedish National Threat Assessment on fentanyl analogues and other synthetic opioids. National Operations Department. https://polisen.se/siteassets/dokument/ovriga_rapporter/fentanyl-analogues-report-english.pdf

[bibr39-14550725221079524] Tollin K. Hammar L. Jonsson A. (2021). Narkotikamarknader—En studie av smuggling, gatuförsäljning, internethandel och köpare [Drug markets: A study of smuggling, street selling, online trade and buyers] (2021:10). The Swedish National Council for Crime Prevention. https://bra.se/download/18.1f8c9903175f8b2aa70f67b/1631001260503/2021_10_Narkotikamarknader.pdf

[bibr40-14550725221079524] Träskman P.-O. (2011). Narkotikabrotten och kontrollen av bruket av narkotika genom straffrättsliga medel [Drug offences and the control of the use of drugs by means of criminal law]. In Olsson B. (Ed.), Narkotika—Om problem och politik (pp. 43–72) [Drugs: On problems and politics]. Nordstedt Juridik.

[bibr41-14550725221079524] Tzanetakis M. Kamphausen G. Werse B. Laufenberg R. (2016). The transparency paradox: Building trust, resolving disputes and optimising logistics on conventional and online drugs markets. International Journal of Drug Policy, 35, 58–68. 10.1016/j.drugpo.2015.12.010 26809972

[bibr42-14550725221079524] Van Hout M. C. Bingham T. (2013). “Silk Road”, the virtual drug market-place: A single case study of user experiences. International Journal of Drug Policy, 24(5), 385–391. 10.1016/j.drugpo.2013.01.005 23465646

